# The introduction of mutations in the wild type coxsackievirus B3 (CVB3) IRES RNA leads to different levels of *in vitro* reduced replicative and translation efficiencies

**DOI:** 10.1371/journal.pone.0274162

**Published:** 2022-10-03

**Authors:** Jawhar Gharbi, Mohammed A. Almalki, Manel Ben M’hadheb

**Affiliations:** 1 Department of Biological Sciences, College of Science, King Faisal University, Al-Ahsa, Saudi Arabia; 2 Virology and Antiviral Strategies Research Unit [UR17/ES30 ViroBiotech], Institute of Biotechnology, University of Monastir, Monastir, Tunisia; Institut Pasteur, FRANCE

## Abstract

Coxsackievirus B3 (CVB3) is a principal causative agent of viral myocarditis, meningitis and pancreatitis. There is no vaccine available for clinical use. It has been demonstrated that the primary molecular determinant of virulence phenotype is located in the 5’ UTR of the viral genome. Translation initiation of CVB3 RNA is directed by the IRES element situated in the 5’UTR. In the present study, we analyse the effects of single point mutations introduced in different positions in the domain V of the IRES RNA of CVB3 wild type. We characterize *in vitro* virus replicative capacitiy and translation efficiency and we test *in vivo* virulence of different CVB3 mutants produced by the introduction of different mutations in the domain V of IRES by site-directed mutagenesis to abolish its structure. Our results demonstrate that all RNA mutants display different levels of decreased replication and translation initiation efficiency *in vitro*. The translation defect was correlated with significant reduced viral titer of mutant particles in comparison with the wild type. When inoculated in mice, mutant viruses were checked for inflammation and necrosis.*In vitro* and *in vivo* Findings strongly suggest that the most attenuated mutant strain could be considered a candidate for live-attenuated CVB3 vaccine.

## Introduction

Coxsackievirus B3 (CVB3) is a human enterovirus (EV)which belong to the *Picornaviridae* family, a large and complex family of small RNA viruses.Enteroviruses infecting humans are assigned to four species, *Enterovirus A* to *Enterovirus D*. Coxsackieviruses B, six serotypes of which are known (CVB1 to CVB6) belong to the *Enterovirus B* species [1]. A positive-stranded RNA molecule that is approximatively 7400 nucleotides long constitutes the CVB3 genome. Recently, a new short upstream open reading frame (uORF) was discovered in some representative members of enterovirus species [2] infecting gut epithelial cells which encodes a small functional upstream protein (UP) in addition to the well-known single ORF (open reading frame) that encodes a polyprotein with 2185 amino-acids that is cleaved during translation by two viral enzymes. This single long open reading frame is flanked by a 5’ Untranslated Region (5’UTR), 742 nucleotides (nt) long and a much shorter 3’UTR which terminates in a poly(A) tract [3] that are highly conserved structured RNA sequences and are important for virus replication and translation [4]. Replication is initiated in the cell cytoplasm by the synthesis of a complementary RNA strand of negative polarity [5]. This double-stranded genome intermediate is subsequently encapsidated by vesicles [6] to produce the replication complex, a membranous double-stranded RNA complex containing virally encoded proteins and cellular factors [7]. Like polioviruses, CVB3 shuts off host cell protein translation [8, 9]. Earlier studies showed that a portion of the 5’UTR could act as "landing pad" for ribosomes, thus enhancing translation of a downstream cistron [10, 11]. This structure, named the Internal Ribosome Entry Segment (IRES), can accept ribosomes even in the absence of a cap 5’ end, shown by IRES-dependent translation of a circularized mRNA molecule. In picornaviruses, IRES elements belong to one of three groups of IRESes, which differ in sequence, secondary structure, and the location of the translational initiation codon. Enteroviruses (EV) and rhinoviruses (RV) contain a type I IRES and the initiation codon is usually 50 to 150 nucleotides downstream from the 3’ end of the ribosome-binding site. The IRES sequence belonging to the 5’UTR of PV has a modular structure in which especially stem-loop domain V regulate replication and translation of the viral genome [10]. Given the critical functional activities of the 5’UTR, it is not surprising that many virulence determinants have been localized there. Mutagenic, chimeric, and deletion studies have confirmed the close structure-function relationship in the 5’UTR. The prevalence and health consequences of enterovirus infection make improved understanding of the 5’UTR structure fundamentally important. Mutations in these sequences affect RNA structures, can have variable effects on molecular mechanisms (replication and translation), and modulate pathogenic properties. It carry out a variety of important roles, it especially mediate RNA-RNA tertiary interactions and serve as a recognition site for interactions between genome RNA and host cell proteins. It has long been known also that tetraloop structural motifs occur frequently in biologically active RNAs. Tetraloops belonging to the IRES domain V of EV carry out a variety of important functions, such a directing correct folding of RNAs with complicated structures, mediating RNA-RNA tertiary interactions and serving as a recognition site for RNA-protein interactions [4].

EVare transmitted mainly by fecal oral route. Infections by CVB are highly prevalent but are usually subclinical or cause a mild flu-like illness. The association of coxsackieviruses with human heart disease is particularly noteworthy, as these viruses are the most common cause of acute viral myocarditis and are involved in as many as 25% of cases in some studies [12]. In recent studies the enteroviruses detected in human myocarditis and cardiomyopathy have been occasionally identified by serotype. This has included coxsackieviruses B2, 3, 4, and 5 and echovirus 9 and 11, all of which belong to the enterovirus B species [13–15]. However, EV infections can be serious with episodes of meningo-encephalitis, myocarditis, paralysis and a fulminant sepsis-like syndrome. Sporadically, a fulminant coxsackievirus infections can result in acute onset of type 1 diabetes without autoimmunity [16]. These viruses are furthermore incriminated as causes of chronic (inflammatory) diseases such as chronic myocarditis [17], chronic pancreatitis [18] and the more common autoimmune type 1 diabetes [19].

The well-studied poliovirus genome serves as the important exemple for how enteroviruses determine a virulence phenotype [reviewed by 20]. During the 1960, Albert Sabin obtained attenuated strains of each poliovirus serotype, which were unable to productively infect and destroy neuronal cells [21]. Each of the three attenuation mutations located in the 5’UTR sequence region which affect the local high-order RNA structure has been linked to a decreased efficiency of translation in cells of neural origin [22, 23]. These mutations were qualified as attenuating mutations. Much less is known about the molecular virulence of other EV, especially CVB. The sites in the CVB3 genome which control expression of the cardiovirulence phenotype have not been exactly mapped to date. It is known that tetraloop structural motifs occur frequently in biologically active RNAs [24]. Tetraloops carry out a variety of important functions, such a directing correct folding of RNA with complicated structures [25], mediating RNA-RNA tertiary interactions among the viral replication [26] and serving as a recognition site for RNA-host cellular protein interactions [27].

CVB3 virus replicates in murine host and induces diseases, which mimic those in humans. The mouse model is useful for the study of EV pathogenesis [reviewed by 28]. Most of the studies on CVB3 experimental murine models make use of intraperitoneal route of infection. However, the natural route of infection in human is the faecal oral one. Oral infection with CVB3 in neonatal, adult and pregnant mice has been reported earlier [29] and documenting a dose-dependent mild infection in adult mice.The virulence of these viruses depends on efficient recognition of the RNA genome by a large family of host proteins and protein synthesis factors, which in turn relies on the three dimensional folding of the first 750 nucleotides of the molecule. Structural and function informations about this region of the genome is needed to assist in the process of vaccine and antiviral development.

The current study reports the effects of single point mutations within different positions in the IRES RNA genome of wild type CVB3. We characterize *in vitro* replicative capacities and translation efficiencies and we test *in vivo* virulence of different CVB3 mutants produced by site-directed mutagenesis.The identification of new sites in the CVB3 genome that are most important to obtain most decreased replication, less efficient translation and non-virulent phenotype in mice are useful for the conception of live-attenuated vaccine candidates.

## Materials and methods

### Ethics statement

The animal model study in this work has been carried out in strict accordance with the recommendations of the Committee of Ethics of the Institute of Biotechnology of Monastir described in their guide for the use of experimentations with animal models (Agreement not needed). The protocol was controlled and supervised by of a member of the committee. Animal experiences were conducted making all efforts to minimize suffering animals during anesthesia.

Mice controls were bred in the Biotechnology Institute of Monastir animal facility. All animal experiments were in compliance with the recommendations of the Committee of Ethics of the institution in a protocol approved and supervised by the committee.

### Mutants (M1-M6) construction by Site-directed mutagenesis

**Fig 1:**
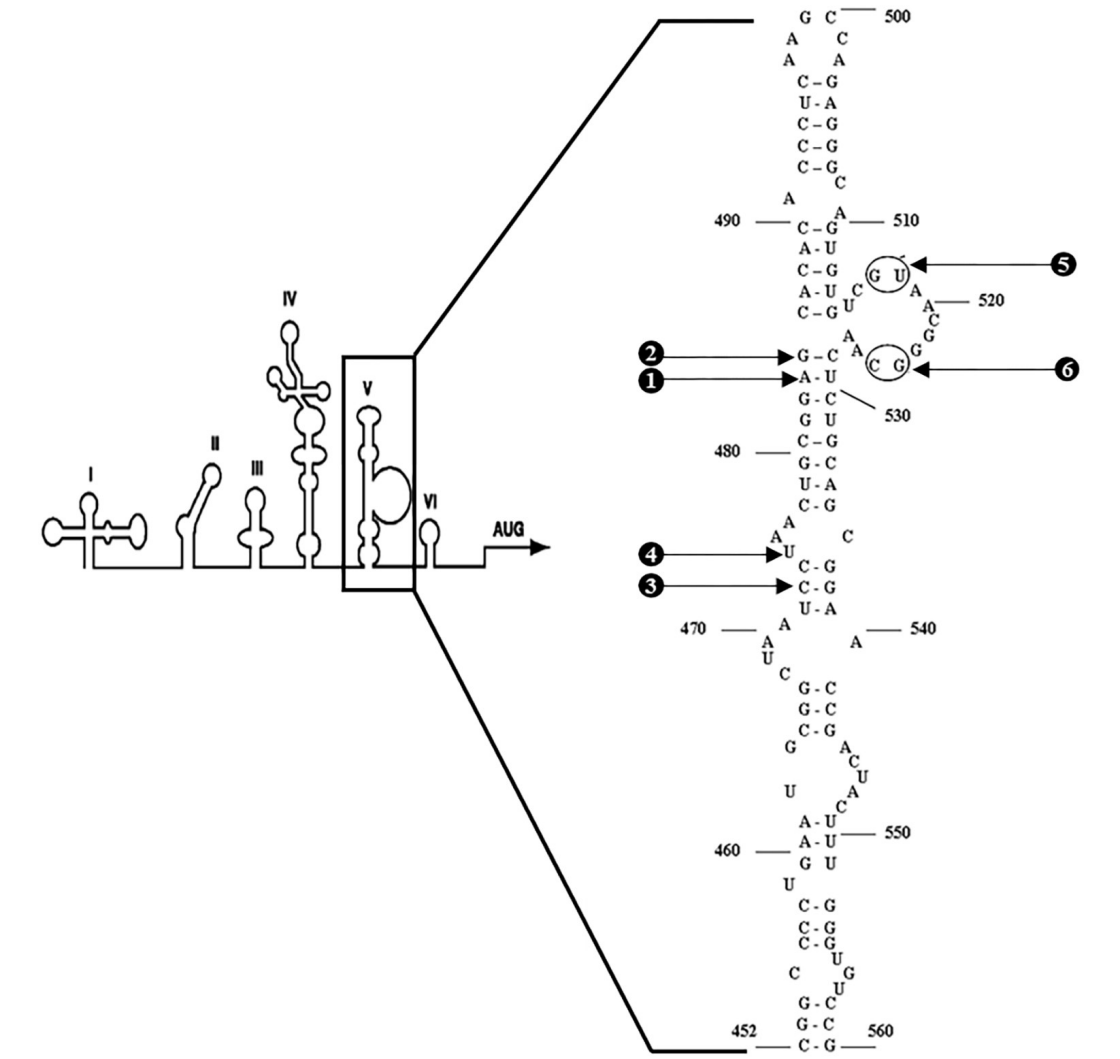
Structure and sequence of domain V of CVB3 IRES. Position of introduced mutations (1–6) on IRES domain V producing CVB3 mutants (M1 to M6) are indicated.

The infectious cDNA clone of the wild type CVB3 Nancy strain, pRibCVB3/T7 obtained from Dr. Willem J. G. Melchers (The Radboud University Medical Centre Nijmegen, The Netherlands). It provided starting material for genetic manipulation. The monocistronic mutant plasmid, ΔpRibCB3/T7 was constructed by deletion of nt 2462 to 6310 of the wild type monocistronic plasmid (pRibCB3/T7). Six different mutations were introduced at different positions of the domain V sequence of the CVB3 IRES segment (Fig 1). Mutants, carrying theses 6-point mutations(single or double mutations) were engineered by oligonucleotide-directed site-specific mutagenesis by using two rounds of PCR with ΔpRibCB3/T7 as a template DNA. The control of stability of all introduced mutation was conducted by automatic sequencing of the domain V of mutant IRES. For the first round of PCR, primers corresponded to nt 28 to 53 (F-PCR1, 5’-CCACAGGGCCCATTGGGCGCTAGCAC-3’) of the CVB3 Nancy cDNA and antisense R-M1 or R-M2 or R-M3 or R-M4 or R-M5 or R-M6 and F-M1 or F-M2 or F-M3 or F-M4 or F-M5 or F-M6 and antisense 1126 to 1106 (R-PCR2, 5’-GGCAACGTCTGGTTGGGTCGG-3’) are used. Primer sequences used to introduce mutations are shown in Table 1. Four from the 6 mutants (M1-M4) carried out 4 single mutations, whereas the other 2 mutants (M5 and M6) carried out double mutations. Mutations M1-M6 correspond respectively to substitutions in the stem loop V of the IRES: A to g at nt 484, g to A at nt 485, C to U at nt 473, U to C at nt 475, g-U to C-A at nt 517–518 and finally g-C to A-g at nt 524-525.Two overlapping mutated PCR products were generated; gel purified, and used as templates for the second round of PCR with primers corresponding to nt 28 to 53 and antisense nt 1126 to 1106 of CVB3 Nancy. The first and the second PCR was carried out for 1 min 20 s at 94°C succeeded by 40 cycles of 40 s at 94°C, 1 min at 63°C, and 1 min at 72°C, followed by 10 min at 72°C. All PCRs conducted with 50 μl volumes containing Pfu polymerase buffer (Invitrogen), 10 mM deoxynucleotide triphosphates (dNTPs), and 1.25 U of Pfu polymerase (Invitogen), 25 pmol of each oligonucleotide (Invitrogen). The *Blp*I/*Eco*RV restriction fragments (nt 291 to 917 of CVB3 Nancy) of the final PCR products replace the counterparts of the ΔpRibCB3/T7. The *Blp*I/*Bgl*II fragments (nt 219 to 2042 of CVB3 Nancy) from the different ΔpRibCB3/T7 mutants, which had been verified by sequencing, were introduced into wild type full-length CVB3 cDNA.

**Table 1:** Primer sequences used to produce RNA IRES Mutants (M1-M6) by the Site-directed mutagenesis method.

Name	Nucleotide Sequences from 5’ to 3’ end	Primer position on CVB3 genome
F-M1	CCTAACTgCgg**g**gCACACACCC	473–494
R-M1	gggTgTgTgC**C**CCgCAgTTAgg	494–473
F-M2	CCTAACTgCggA**A**CACACACCCTCAAgCC	473–501
R-M2	ggCTTgAgggTgTgTg**T**TCCgCAgTTAgg	501–473
F-M3	CCTgAATgCggCTAAT**T**CTAACTgCggAgC	457–486
R-M3	gCTCCgCAgTTAg**A**attagCCgCATTCAgg	486–457
F-M4	CCTgAATgCggCTAATCC**C**AACTgCggAgC	457–486
R-M4	gCTCCgCAgTT**g**ggATTAgCCgCATTCAgg	486–457
F-M5	ggCAgTgTgTC**CA**AACgggCAACTCTgCAgCgg	506–538
R-M5	CCgCTgCAgAgTTgCCCgTT**Tg**gACACACTgCC	538–506
F-M6	ggCAgTgTgTCgTAACgg**Ag**AACTCTgCAgCgg	506–538
R-M6	CCgCTgCAgAgTTAg**CT**gTTCAgACACACTgCC	538–506

### *In vitro* RNA transcription

For transfections, purified plasmids derived from wild type full length and mutants of pRibCB3/T7 were linearized with *Sal*I (nt 7435 of pRibCB3/T7). *In vitro* transcription was carried out as described previously [30]. The produced RNA quantity were measured using spectrophotometer. The obtained RNA was used for transfection without further purification.

*In vitro* translation assays were realized as described previously [26]. RNA quality was evaluated through 0.8% agarose gels electrophoresis and quantity measured by spectrophotometer.

### Hela cells transfections

Sub-confluent monolayers HeLa cells grown in DMEM (Gibco BRL) with 7% FBS (Gibco BRL) were used for transfection with DEAE-dextran as described previously [30]. Control HeLa cells were mock transfected without RNA or with DMEM only. HeLa cells were then harvested when total cytopathic effect (CPE) was observed. Original virus stock was clarified using centrifugation. Titers of produced virus were determined by the classical method of Reed and Munch.

Virus stocks of mutants and wild type were produced on monolayers HeLa cells at the same multiplicity of infection of 30 TCID_50%_ as described previously [30, 31]. All introduced mutations were controlled by automatically sequencing in virus stocks.

### *In vitro* translation

*In vitro* translation reactions were carried out with standard rabbit reticulocyte lysates (RRL) supplemented with HeLa cell S10 extracts prepared and treated with micrococcal nuclease. All reactions contained 50% (vol/vol) RRL (Flexi RRL system; Promega), 417 μCi of [^35^S] methionine/ml (>1,000 Ci/mmol; Amersham), and 80 μM unlabeled amino acids (except for Leucine and Valine, which were present at 120 μM, and Methionine, which was omitted). Added MgCl_2_ and KCl were optimized on the bases of the RRL used from 0.2 to 1.2 mM and 50 to 100 mM, respectively. Concentrations of HeLa cell extracts were varied from 0 to 20% (vol/vol), and that of H100 buffer (10 mM Hepes-KOH [pH 7.5], 1 mM MgCL_2_, 0.1 Mm EDTA, 100 mM KCl, 7 mM β-mercaptoethanol) was varied from 33 to 13% (vol/vol) of the final reaction volume. Reactions were programmed with uncapped mRNAs at concentration of 10 μg/ml, and essay reaction mixtures were incubated for 1h at 30°C. Reactions were stopped by adding stop RNase solution and incubated for 10 min at room temperature before adding of blue solution (80% bromophenol blue, 20% β-mercaptoethanol). Translation products were analyzed by sodium dodecyl sulphate polyacrylamide gel electrophoresis by using gels containing 15% (wt/vol) polyacrylamide. Quantification of translation of the viral polyprotein before its cleavage was carried out by densitometry of autoradiograms by using NIH image software (Image J 1.34s), with multiple exposures of each radiogram to ensure that the linear response range of the film was respected.

### Virus single-step growth cycles

Virus single-step growth cycles in HeLa monolayer cells were performed at 37°C as described previously [30, 31]. Twenty-four-well plates with 2 x 10^4^ cells per well were inoculated with the CVB3 wild type or with one of produced mutants at the same multiplicity of infection of 10 TCID_50%_. Transfected plates were frozen at different times of post infection (0, 3, 5, 7, 10, 15 and 20 h). Virus titers of each mutant and wild type were determined by Reed and Munch method on HeLa cells using 96 well plates.

### Virulence assays in mice

Female Swiss mice (3 to 4 weeks old) were purchased from the Pasteur Institute of Tunis, Tunisia. All mice were divided separately in 3 groups and rested until body weights were between 12 and 20 g per mouse. Mice were inoculated by oral route using a sterile polyethylene tube and syringe with 5 x 10^5^ TCID_50_ of either CVB3 viruses (mutants and wild type viruses) previously titrated on HeLa cell monolayers. Control mice (uninfected mice) obtained 0.3 ml of BME medium only. Mice controls were bred in the Biotechnology Institute of Monastir animal facility. All animal experiments complied with the recommendations of the Committee of Ethics of the institution in a protocol approved and supervised by the committee. The protocol was controlled and supervised by of a member of the committee. Animal experiences were conducted with making all efforts to minimize suffering animals during anesthesia.

Mice were sacrificed every day from 0 to 21 days post inoculation. Heart and pancreas tissues were fixed in formalin for paraffin embedding. Serial 3–5 μm thick sections of different organ tissues were stained with eosin and hematoxylin and examined for the presence of inflammatory lesions by light microscopy.

### Statistical analysis

Student’s t test was used to establish statistical significance of three (n = 3) replicated experiments (*P*< 0.05) using Graphpad Prism 6 Version 6.02.

## Results

### The generated M1-M6 mutants growth as well as CVB3 wild type but with different level of reduced replicative efficiencies

**Fig 2:**
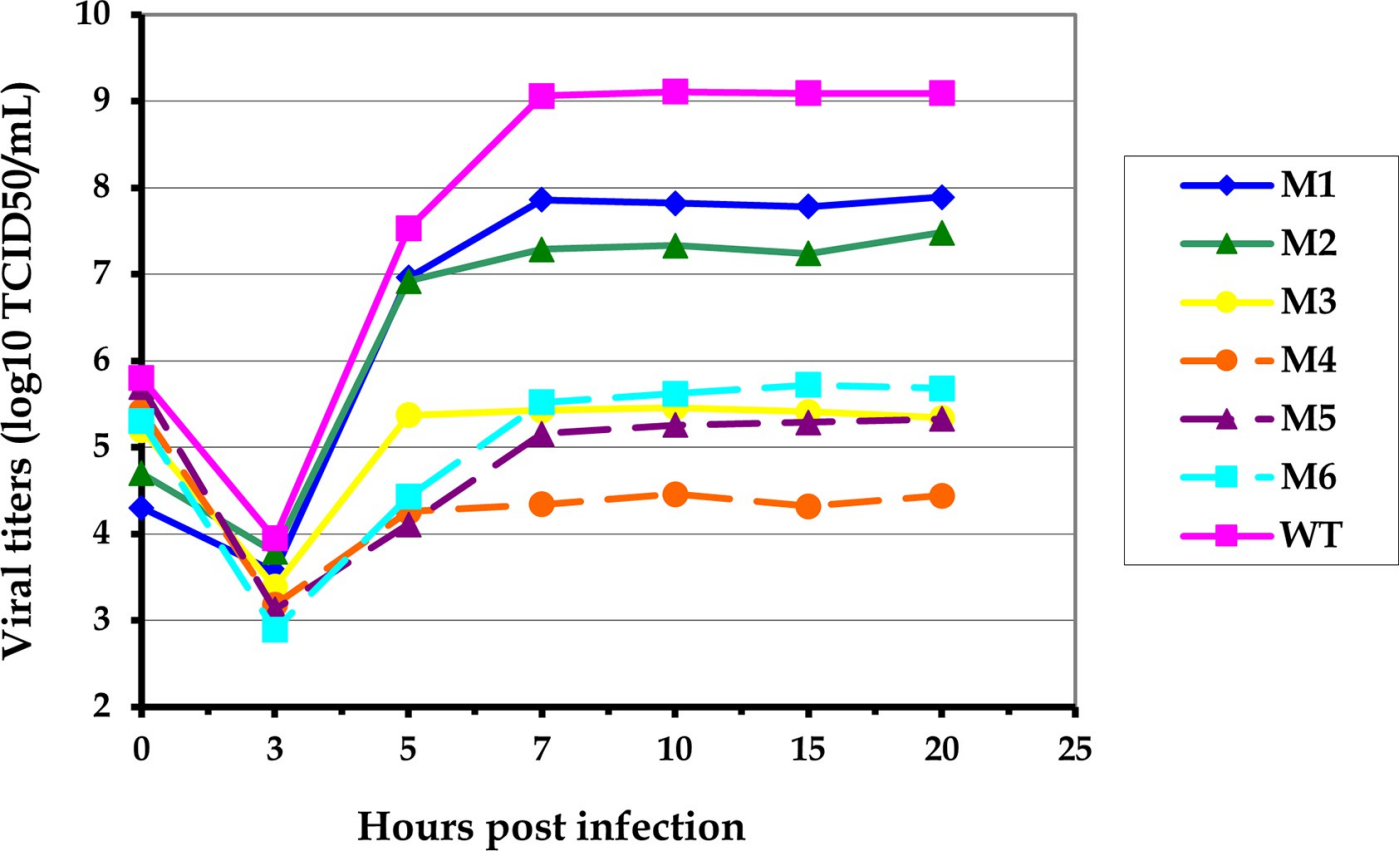
One-step growth cycle of CVB3 wild type and M1-M6 mutant viruses in HeLa cells. Cells were infected at a MOI of 10 TCID_50_/cell at 37°C and 5% CO_2_. Samples were analysed for infectious virus particle formation at the indicated times by determining titers using Reed and Munch method on HeLa cell monolayers harvested by freezing at specific times shown. Experiments were performed in triplicate. p.i., post infection. The data shown are the mean +/- SD from three replicated experiments (n = 3). *P*< 0.05 compared *vs* control, Student’s t test.

The six mutant RNAs (M1, M2, M3, M4, M5 and M6) or RNA encoding wild type of CVB3 were transfected into HeLa cells, which are known to support productive CVB3 infection. Seventy two hours later, supernatants were stocked and infectious virus titers were determined by Reed and Muench method. Viral supernatant from cells transfected with the M3, M4, M5 and M6 RNAs yielded viruses with almost1 log decreased titers, suggesting that mutations introduced within the stem-loop V of the IRES of these mutants were attenuating. However, infectious viruses of M1 and M2 mutants were detected with titers comparable to that of wild type CVB3, suggesting that the virus mutants were not severely disabled in tissue culture (Fig 2).

All mutant virus stocks were compared to wild type of CVB3 by the use of single-step growth cycles. Stocks of wild type of CVB3 and mutant viruses of similar titers, were used to infect HeLa cells at an MOI of 10 (10 viral particles infect one cell) and samples were harvested at 3, 5, 7, 10, 15 and 20 h p.i. and then titred by Reed and Munch method. As demonstrated in Fig 2, the growth kinetics of the M1 and M2 mutant viruses were almost similar to that of wild type of CVB3. the first distinguishable increase in titers was observed at 3 h p.i.. At this and most subsequent time points, CVB3 wild type titer was higher of 1 to 1.5-fold than the two mutants M1and M2. Therefore, we conclude that M1 and M2 CVB3 mutants present the mutations that lead to a less attenuated replicative capacities comparing to the CVB3 wild type. Interestingly, the two mutants are always and continue to replicate in tissue culture.

However, M3, M4, M5 and M6 CVB3 mutants were severely handicapped in HeLa cell monolayers, as demonstrated by their single-step growth cycles showed in Fig 2. Indeed, in the same conditions of culture, wild type CVB3 virus produced 4 to 5-fold more infectious virions per cell than all others mutant viruses. The defective nature of M4 was particularly marked at the same temperature, where it replicate and produce infectious viral particles but with 5-fold less titer than the wild type of CVB3. Again, results of single-step growth demonstrated that all other mutant viruses continue toincrease in titer over time in the same cell culture as well as the wild type virus.

### *In vitro* translation of mutant and wild type RNA demonstrated different levels of translation efficiency

**Fig 3:**
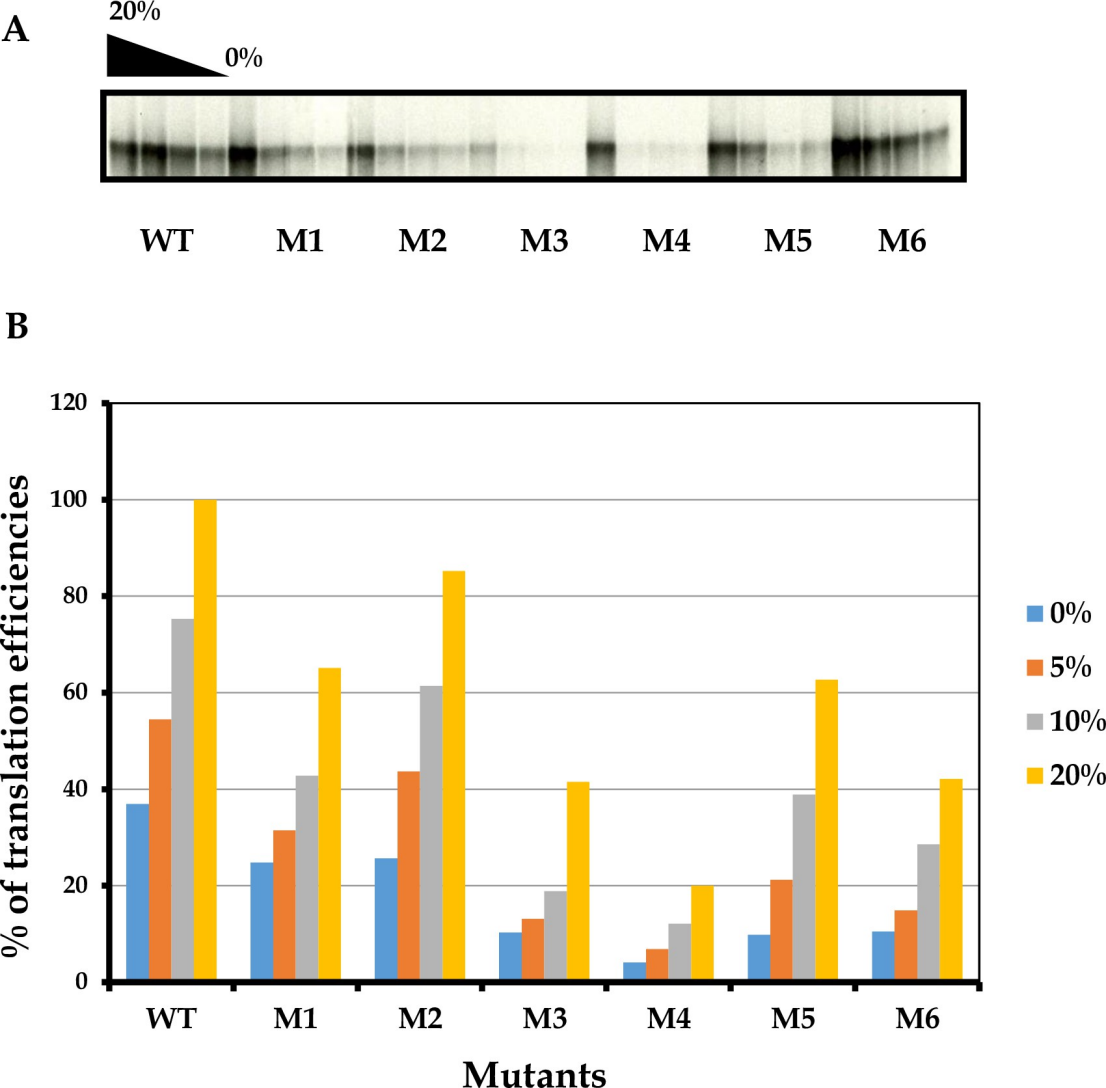
**(A)** Polyprotein products of *in vitro* translation of wild type and M1-M6 mutants of CVB3 in cell-free reaction mixtures supplemented with decreasing S10 HeLa cell extracts (20% to 0%). Transcript RNAs derived from wild-type CVB3 and its mutants were translated and analyzed in HeLa cell extracts as described in material and methods. **(B)** *In vitro* translation efficiencies of CVB3 wild type and M1-M6 mutants. Densitometric quantifications of synthetic wild type and mutant polyproteins revealed on dried SDS-polyacrylamide gel. The percentages indicated are those of S10 extract. The data shown are the mean +/- SD from three replicated experiments (n = 3). *P*< 0.05 compared *vs* control, Student’s t test.

CVB3 RNA mutant transcripts were translated *in vitro* in a RRL supplemented with different increasing concentrations of S10 cytoplasmic extracts of HeLa cells (0 to 20%) and with a 10 μg/ml as RNA template in order to investigate the translation efficiencies of the different produced RNA mutants. The quantification of all polyproteins generated by each IRES RNA mutants was conducted by the measure of the intensity of bands obtained by SDS-PAGE (Fig 3A) using the NIH image program. Results of the measurement of the intensity of bands are shown in Fig 2B. Quantities of viral polyproteins of the wild type and of the different RNA mutants were very different, indicating that introduced point mutations within the IRES stem-loop V of the genome of CVB3 leads to different effects on *in vitro* translation efficiency of each mutant.

Obtained results showed a decreasing level in translational efficiency of all RNA mutants and it is observed upon supplementation of translation extracts with such S10 cytoplasmic extracts. Mutants M1 and M2 reach respectively 65% and 85% of the translation efficiency to direct translation initiation of the CVB3 wild type. However, the M3 (41.51%) and M4 (20%) RNA mutants, a little translation was detected. Moreover, their RNA mutants showed low sensitivity to the addition of non-canonical factors, which prove that the 2 mutants were affected in their translation. M5 RNA mutant directed translation at low efficiency (62.67% compared with the level of wild type). In addition, M6 RNA mutant revealed a dramatic decrease in translation efficiency with the same conditions. M6 RNA mutant was translated only at 42.13% comparing with the wild type RNA (Fig 3B).

### CVB3 mutants leads to different degrees of organ inflammations when are inoculated to mice

**Fig 4:**
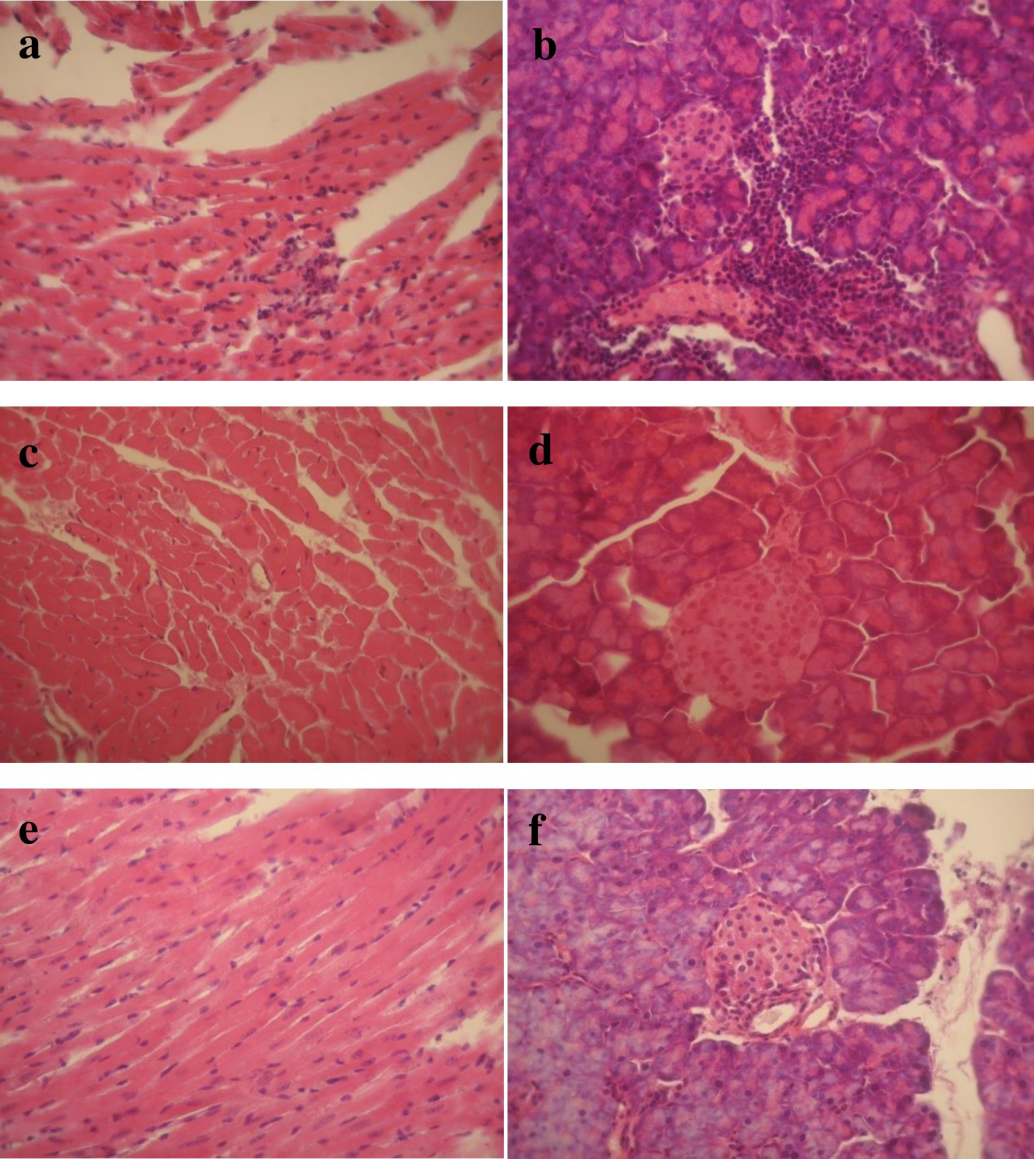
Histology of hearts and pancreas of Swiss mice at day 10 after oral inoculation with CVB3 wild type and mutant viruses as described previously [26]. Shown are murine heart (a) and pancreas (b) tissues stained with hematoxylin and eosin from mice inoculated with CVB3 wild type, (c, d) heart and pancreas from a control uninfected mice and (e, f) heart and pancreas from mice inoculated with M3 virus (all 4 mutant viruses induced a similar lack of pathology in mice, S1 Fig). Lymphocyte infiltrations are shown in each organ tissues. Experiments were performed on three mice for each mutant and for each day post inoculation.

In order to determine whether the attenuated replication and translation of CVB3 mutants in HeLa cell corresponded to an attenuation of the virulent phenotype of the wild type CVB3 strain, mice we inoculated by oral route (the natural route of contamination by CVB3). Heart and pancreas tissues were evaluated for evidence of pathologic damage. Mice tissues stained with hematoxylin and eosin in thick sections were examined by light microscopy. Tissue damages of heart and pancreas due to virulent CVB3 replication has been demonstrated previously to be evident at 10 days post inoculation [29]. Results of histology examination showed that from 10 days post inoculation, CVB3 Nancy strain provokes widespread inflammatory lesions and significant extension of infiltration and necrosis area (pancreatitis and myocarditis) in heart and pancreas muscles of mice (Fig 4A and 4B). Hearts and pancreas of mice inoculated with M3-M6 (Fig 4E and 4F) mutant viruses appeared normal and indistinguishable from heart from mock-infected mice (Fig 4C and 4D). At day 21 post inoculation, there was still no evidence of lesions in organs tissues of mice inoculated by CVB3 mutants. Results on mice model demonstrate that M3-M6 mutant were considered to be with attenuated phenotypes.

## Discussion

The IRES sequence belonging to the 5’UTR of PV has a modular structure in which especially stem-loop domain V regulate replication and translation of the viral genome [32]. Mutations in these sequences affect RNA structures, can have variable effects on molecular mechanisms (replication and translation), and modulate pathogenic properties [33]. Attenuated phenotype showed in Sabin strains (Sabin 1, Sabin 2 and Sabin 3) was essentially caused by the defect of translational efficiency of these strains in neural cells [20]. It has been known that the domain V of IRES containing tetraloop structural motifs occur frequently in molecularly active RNAs. This domain carry out a variety of important roles, it especially mediate RNA-RNA tertiary interactions and serve as a recognition site for interactions between genome RNA and host cell proteins. The most significant new insights revealed recently by Mahmud et al., (2019) [34] into enteroviral 5’ UTR structure were found in domain V. These findings were particularly important given the role of domain V in ITAF binding, canonical initiation factor binding, ribosome binding, and virulence. This new model adds roughly 60 residues to the length and substantially rearranges domain V into a complex junction loop. This rearrangement brings a number of functionally important elements into close structural proximity. The predicted structure of domain V is highly complex and can be broken into four stem-loop regions that are connected by two multiloops. Sabin vaccine strains of poliovirus have functionally deleterious mutations within this domain. Sabin-like mutations of the CV-B3 5’ UTR have debilitating effects on viral replication and translational efficiency [30, 31].

Partially attenuated CVB3 strain was obtained by long-term passage of a virulent CVB3 strain in host cell culture [35]. A single mutation at nt690 in the region of the 5’UTR of this CVB3 virulent strain genome was identified to be irrelevant of the less virulence phenotype. The comparison of RNA genome of non-cardiovirulent CVB3/0 strain and wild type RNA genome [36] revealed two important regions in the 5’UTR as well as 8 amino acid changes which play an important role as a function of cardiovirulence phenotype [3].

5’UTR of EV is a highly ordered structure among different species and serotypes. Because it is considered as the central of both transcription and translation of the viral genome, our main objective of this present study was the study of the relationship between the IRES stem-loop V of CVB3 and the degrees of replication and translation efficiencies. More previous study demonstrated that in poliovirus 5’UTR, a single nucleotide mutations defined by nt 472 to 481 is significantly and perturb RNA structures [37]. These 5’UTR transitions considered as attenuated have been linked to the decreasing of the translational efficiencies of mutant strains relative in comparison with wild type [22, 23]. The 5’UTR of CVB3 has a modular organization in which the IRES regulate RNA replication and translation, respectively [3, 38, 39]. The present work shows that different mutations at different positions in the sequence of IRES domain V of CVB3 affect the biological characteristics of the virus at different levels. We report then that this domain V from the IRES plays an important function that so far escaped detection.

Our results were based on phenotype analyses of 6 CVB3 mutants whose genomes harbour one to six nucleotide substitutions (A to g at nt 484, g to A at nt 485, C to U at nt 473, U to C at nt 475, gU to CA at nt 517–518 and gC to Ag at nt 524–525) compared to the genome of wild type CVB3 strain. Secondary structure prediction of the region surrounding the IRES domain V (nucleotide position 470 to 530) of all mutants and the wild type, using mFOLD prediction software reveals that there is no alteration in the RNA structure in mutants comparing to the wild type. Properties growth of these six CVB3 mutants and wild type CVB3 were investigated by carrying out a one-step growth cycles study in HeLa cells. The M1 and M2 mutants displayed respectively a 1 to 1.5 log unit reduction of viral particles production in HeLa cells. The other 4 CVB3 mutants showed reduction of their replication. They displayed a 3 to 4.5 log unit reduction of infectious particles production in HeLa cells comparing with CVB3 wild type.

A previous work conducted by Bhattacharyya and Das [38] observed a decreasing in translation efficiency of viral RNA containing attenuating mutations compared with that from virulent wild type viruses. Thus, suggesting that viral translation plays an important function in the attenuation of CVB3. The goal of this study is to determine the levels of translation efficiencies directed by the mutated RNA of the 6 CVB3 mutants *in vitro* in a cell-free system and to correlate it to the viral production capacities. The cellular factors necessary for EV translation are present in sufficient amounts in HeLa cell S10 extract but not in RRL used in our experience. Thus, the *in vitro* system is useful for the study of the cap independent translation and the identification of cellular factors necessary. Indeed, using this system, the M1 and M2 mutated RNA templates directed translation as efficiency as wild type RNA. However, a significant reduction in the translation efficiency of the M3, M4, M5 and M6 RNA was observed in *in vitro* translation experiments. Our results of *in vitro* translation suggest that the RNA of M1 and M2 CVB3 mutants interact with translation machinery present in HeLa cells with the same manner of that of the wild type RNA template. However, the four other mutants, especially the M4mutant present a pronounced level of defect in translation that is more important than those of the M1 and M2 mutants. Even if the nucleotide changes in domain V of the M3, M4, M5 and M6 RNAs may not influence the overall RNA structure of the IRES element, the altered nucleotide sequences of these four mutants stop the RNA interaction to proteins involved in translation initiation. This RNA-protein stopped interaction may then be responsible for the reduced levels of viral protein synthesis observed.

The molecular genetic information that determine the CVB3 pathogenic phenotype have been remained unsolved. Results of the present study support our originally hypothesis witch suppose that the CVB3 5’UTR of the genome comprises one or more primary genetic determinants witch influence strongly the biological properties of CVB3. Worldwide poliomyelitis vaccination effort to eradicate wild polioviruses moves to completion, it is important to evaluate alternative HEV other than poliovirus to fill this gap. Thus, it is important to explore methodology to attenuate HEV like CVB3 for the use as potential vaccine candidates. In the present study, we demonstrate that introduced mutations within the domain V of the IRES of a virulent strain of CVB3 leads to different avirulent mutant viruses. Our produced mutated viruses leads to different levels of attenuation in replication on cell cultures, *in vitro* translation in standard RRL lysate and in prolonged infection in mice model. Indeed, 10 days after oral inoculation, CVB3 wild type induce widespread inflammatory lesions in heart and pancreas in infected mice, whereas M5, M6 and especially M4 mutant viruses demonstrate no evidence of inflammatory disease neither in heart or pancreas of orally inoculated mice. Results of our *in vivo* study were comparable to those obtained for other well-characterized, attenuated strains of CVB3 [39]. Additional investigations are necessary to clarify the real molecular mechanism of attenuation and to determine the vaccine capacities of these attenuated mutants by studying especially the extent of replication of these mutant viruses in the mouse tissues and the developing of anti-CVB3 antisera and immunoreactivity to challenge in animal model.

## Supporting information

S1 Fig Histology of hearts and pancreas of Swiss mice at day 10 after oral inoculation with M4, M5 and M6 CVB3 mutant viruses. Shown are murine heart (a) and pancreas (b) tissues stained with hematoxylin and eosin from mice inoculated with CVB3 M4 mutant, (c-d) heart and pancreas from mice inoculated with CVB3 M5 mutant and (e-f) heart and pancreas from mice inoculated with M6 virus.
(DOCX)
