# Supplementary material for: The introduction of mutations in the wild type coxsackievirus B3 (CVB3) IRES RNA leads to different levels of in vitro reduced replicative and translation efficiencies
Source: PLoS One. 2022 Oct 3;17(10):e0274162. doi: 10.1371/journal.pone.0274162 (PMC9529112; doi:10.1371/journal.pone.0274162)
Supplement: S1 Fig — Shown are murine heart (a) and pancreas (b) tissues stained with hematoxylin and eosin from mice inoculated with CVB3 M4 mutant, (c-d) heart and pancreas from mice inoculated with CVB3 M5 mutant and (e-f) heart and pancreas from mice inoculated with M6 virus. (DOCX) [file pone.0274162.s001.docx]

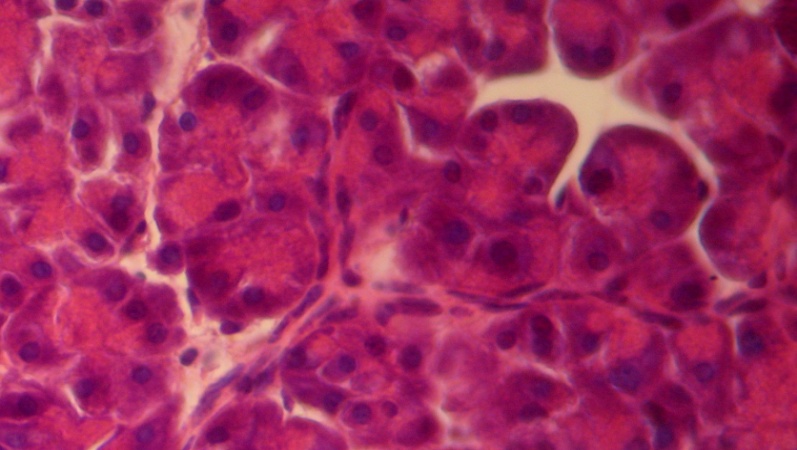

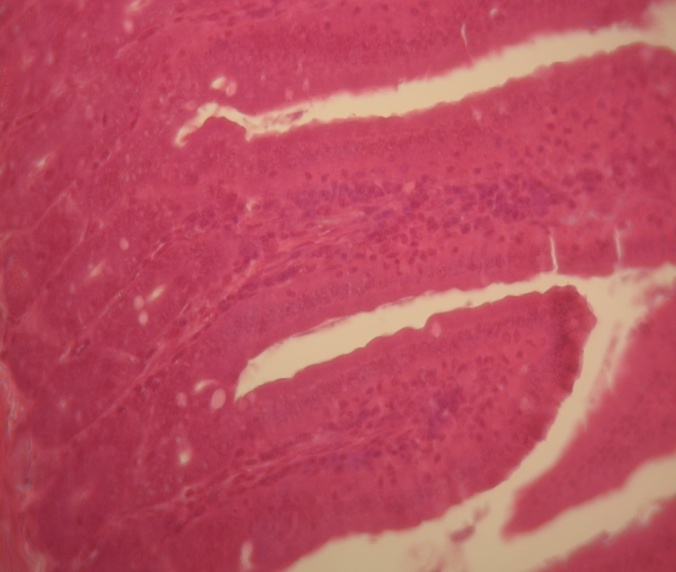

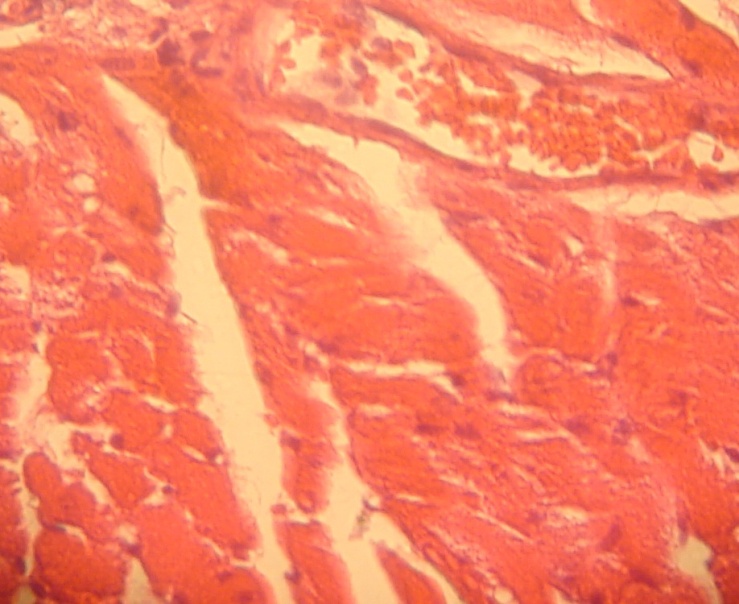

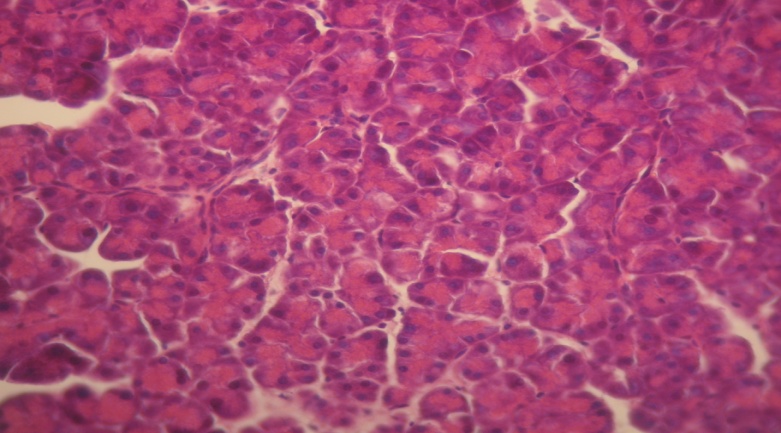

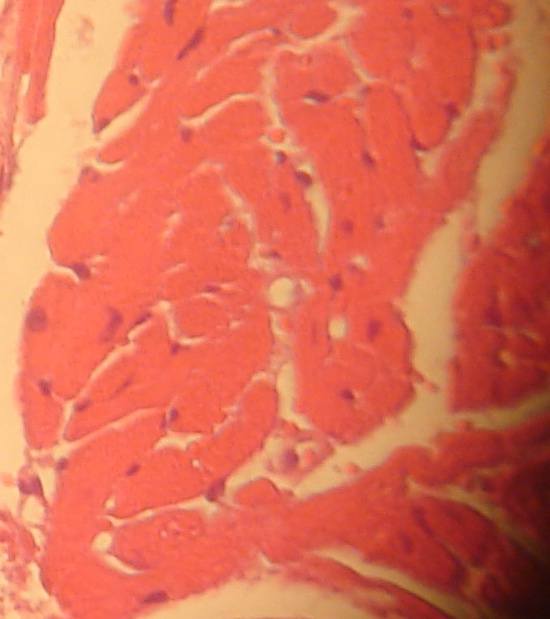

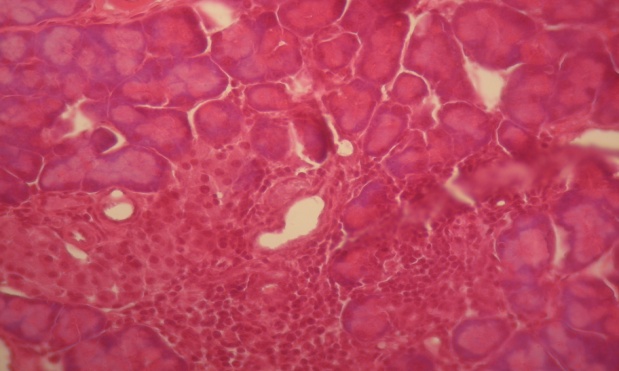


**b**

**f**

**e**

**d**

**c**

**a**

**Supplementary Figure :**

Histology of hearts and pancreas of Swiss mice at day 10 after oral inoculation with M4, M5 and M6 CVB3 mutant viruses

Shown are murine heart (a) and pancreas (b) tissues stained with hematoxylin and
eosin from mice inoculated with CVB3 M4 mutant, (c-d) heart and pancreas from mice inoculated with CVB3 M5 mutant and (e-f) heart and pancreas from mice inoculated with M6 virus.
